# Amplification of a transgene within a long array of replication origins favors higher gene expression in animal cells

**DOI:** 10.1371/journal.pone.0175585

**Published:** 2017-04-12

**Authors:** Kiwamu Ohsaki, Yusuke Ohgaki, Noriaki Shimizu

**Affiliations:** Graduate School of Biosphere Science, Hiroshima University, Higashi-hiroshima, Hiroshima, Japan; Center for Cancer Research, UNITED STATES

## Abstract

Plasmids with both a mammalian replication initiation region (IR) and a matrix attachment region (MAR) are spontaneously amplified in transfected cells, and generate extrachromosomal double minute (DM) or chromosomal homogeneously staining region (HSR). We previously isolated the shortest core IR (G5) required for gene amplification. In this study, we ligated the G5 DNA to create direct or inverted repeats, mixed the repeats with an expression plasmid, and transfected the mixture into human COLO 320DM or hamster CHO DG44 cells. Consequently, we found that the transfected sequence generated DMs or HSR where, surprisingly, the plasmid sequence was embedded within a long stretch of G5 sequences. The amplified structure from the direct G5 repeats was stable, whereas that from the inverted repeats was not. The amplification might be explained by the efficient replication/multimerization of the G5 repeat and recombination with the co-transfected plasmid in an extrachromosomal context. The product might then be integrated into a chromosome arm to generate a HSR. The expression from the plasmid within the long G5 array was much higher than that from a simple plasmid repeat. Because G5 is a core IR that favors gene expression, a long array of G5 provides an excellent environment for gene expression from the embedded plasmid.

## Introduction

Amplification of specific genes in a cell plays a pivotal role in genome evolution [1] and human cell malignant transformation [2]. Furthermore, gene amplification is widely used for the efficient production of recombinant proteins, e.g., biopharmaceuticals [3]. We previously developed a novel and efficient method to amplify a gene of interest in mammalian cells [4]. Specifically, a plasmid bearing a mammalian replication initiation region (IR) and a matrix attachment region (MAR) multimerizes to form a large circular molecule where the initial plasmid is arranged as tandem direct repeats [5]. Such large circular molecules can be maintained as an extrachromosomal episome or a double minute (DM) chromatid, which are known as sites of gene amplification in human cancer cells [5]. If the episome or DM is integrated into a chromosome arm, the plasmid repeat efficiently initiates the breakage-fusion-bridge (BFB) cycle, which generates a chromosomal homogeneously staining region (HSR) [6]. HSRs are also known as sites of gene amplification in cancer cells. Interestingly, any DNA co-transfected with the IR/MAR plasmid can be co-amplified in the cells [5], which might reflect frequent intermolecular recombination at the extrachromosomal site. IR/MAR gene amplification provides an efficient method for recombinant protein production [7–9].

The extrachromosomal replication and maintenance of the IR/MAR plasmid, at least during the initial stage after transfection into the cell, appears to play a central role during the amplification, and the IR/MAR might function as a replicator. A replication fork barrier sequence, if placed in the IR/MAR plasmid, blocks gene amplification in an orientation-dependent manner [5]. We previously dissected the IR from *DHFR*, c-*myc*, and ß-globin loci, and obtained the shortest required sequence (core IR) that supports gene amplification [10, 11]. Such sequences are always enriched with several sequence elements that are required for replication initiation. G5 is one such core IR from the ß-globin locus [11].

A palindromic sequence is frequently found in the replication initiation site (reviewed in [12]). The palindrome forms a cruciform structure, and the cruciform-binding protein may regulate the replication initiation [13, 14]. The palindromic sequence might also trigger the gene amplification [15–17]. Therefore, we initially wanted to know the effect of inverted or direct repeats of core IR (G5) on gene amplification. However, cloning such repeats using a plasmid vector in bacterial host cells is extremely difficult. Therefore, we made the repeats by cell-free ligation, and co-transfected them with the plasmid DNA. This experiment serendipitously resulted in the discovery of a unique method that amplifies the gene of interest within a long stretch of the core IR, providing an artificial environment that promotes gene expression and avoids gene silencing. This may provide a powerful method for recombinant protein production, and may also provide a tool to analyze replication initiation.

## Materials and methods

### Plasmids

**Fig 1:**
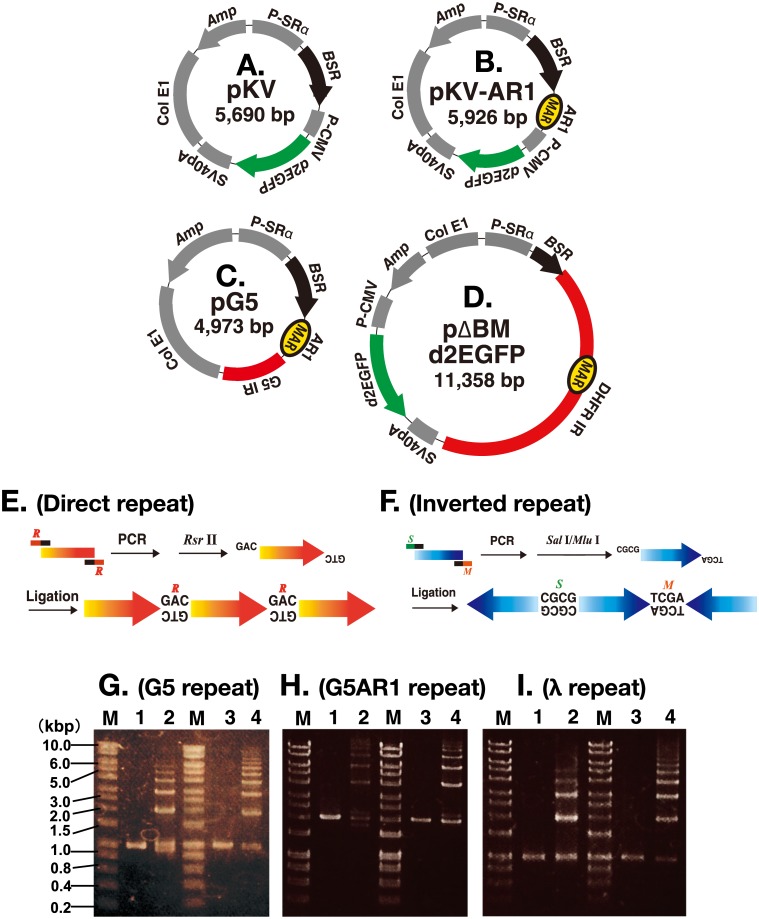
Preparation of repeat DNA and construction of plasmids. (A to D) Structure of the plasmids used in this study. (E and F) Direct or inverted repeat DNA was prepared by PCR amplification, digestion with *Rsr* II (R) or double digestion with *Sal* I (S) and *Mlu* 1 (M), respectively, followed by ligation. (G to I) G5 (G), G5AR1 (H), or a portion of λ (I) DNA before (lanes 1 and 3) and after ligation to direct repeats (lane 2) or inverted repeats (lane 4). DNA was separated by agarose gel electrophoresis. M; molecular weight marker.

pKV (Fig 1A) contained blasticidin resistance (*BSR*) and destabilized enhanced GFP (*d2EGFP*). It was constructed by removing the hygromycin expression cassette from pSFV-V [4] by *Bam* HI/*Nru* I digestion, followed by insertion of the *d2EGFP* expression cassette, which was derived from pCMV-d2EGFP [18], using the In-Fusion HD Cloning Kit w/Cloning Enhancer (Clontech). pKV-AR1 (Fig 1B) was similar to pKV, but contained the AR1 sequence from an Ig k intron that showed strong *in vitro* MAR activity [19]. It was constructed by cutting the multiple cloning site of plasmid pTV-MCS with *Bam* HI [11], followed by insertion of the *d2EGFP* expression cassette as described above. Construction of pG5 (Fig 1C) [11] and pΔBM d2EGFP (Fig 1D) [20] has been described previously.

### Preparation of repeat DNA

Preparation of direct or inverted repeat DNA is diagrammatically shown in Fig 1E and 1F. The G5 sequence (971 bp in pG5 plasmid [11]), the G5-AR1 sequence (2,039 bp in pTVRepP [11]), or position 1,001–1,972 (971 bp) of λ-phage (NCBI NC001416) was PCR amplified using pG5, pTVRepP, or λ-phage DNA as a template, respectively. For primer sequences, see S1 Table. Each PCR primer had a 20 nt sequence specific to the target, which was followed by a 13–14 nt sequence with a restriction enzyme recognition site at the 5’ end. For the preparation of inverted repeat DNA, either a *Sal* I or *Mlu* I site was included in the right or left primer, respectively. *Sal* I and *Mlu* I are conventional type II enzymes. For the preparation of direct repeats, a *Rsr* II site was included in both left and right primers. *Rsr* II is a type I restriction enzyme, which cuts non-palindromic sequences. After PCR amplification using these primer sets, KOD-Plus Neo (Toyobo Co.) DNA polymerase, and a template DNA, the products were digested by the restriction enzyme that cuts at each primer sequence. The DNA was purified using NucleoSpin^®^ Gel and PCR Clean-up (MACHEREY-NAGEL Co.) and ligated using Ligation high Ver.2 (TOYOBO Co.). The ligated DNA was purified by phenol/chloroform extraction and ethanol precipitation, and was used for cell transfection. The electrophoretic analysis of the preparation is shown in Fig 1G to 1I.

### Cells, culture, transfection, and selection

The human colorectal carcinoma COLO 320DM cell line (ATCC CCL 220) was cultured in RPMI 1640 medium (Nissui Pharmaceutical Co. Ltd.) supplemented with 10% FCS. The Chinese hamster CHO DG44 cell line was obtained as described [8], and cultured in Ham’s F-12 medium (Nakarai Tesque Inc.) supplemented with 10% FCS. For transfection, COLO 320DM cells (2x106 cells) were suspended in 1 ml fresh medium and transfected with 2 μg DNA and 7 μl GenePORTER^™^2 Transfection Reagent (Genlantis Co.) in a 3 cm dish, according to the manufacture’s recommended protocol. CHO DG44 cells were plated 24 hours before transfection at the density of 8 x 105 cells/3 cm dish. They were transfected with 4 μg DNA and 10.5 μl Lipofectamine 2000 Reagent (ThermoFisher Scientific Co.). Co-transfection of plasmid DNA and repeat DNA usually employed a mixture of equal weight of each DNA. The cells were selected by 5 μg/ml blasticidin (Funakoshi Co.) starting 2 days after transfection, and the colonies were suspended and subcultured after 3 weeks (COLO 320DM cells) or 2 weeks (CHO DG44 cells).

### Fluorescence In Situ Hybridization (FISH) and flow cytometric analysis

Metaphase chromosome spreads were prepared by a standard protocol [21]. Chromatin fibers were prepared according to our previously published protocol [5], which was developed based on the preceding protocol [22]. DIG-labeled plasmid probes were prepared from pG5 or pKV-AR1 plasmid DNA using the BioPrime DNA Labeling Kit (Invitrogen) and 10× DIG DNA Labeling Mixture (Roche). Biotin-labeled G5 probe was prepared by PCR amplification of the G5 sequence (966 bp) in the pG5 plasmid using the primer set used for the preparation of repeat DNA (see above) and biotin labeling of the product using the Biotin PCR labeling kit (Promokine). The probe was hybridized to metaphase chromosome spreads, and hybridized DIG- and biotin-labeled probes were detected using anti-Digoxigenin-Fluorescein Fab fragments (Roche) or Streptavidin Alexa Fluor 594 conjugate (Invitrogen), respectively. For the flow cytometric analysis to evaluate *d2EGFP* expression, the cells were resuspended in phosphate buffered saline and analyzed using FACS Calibur (Becton Dickinson Co.).

### Quantification of gene copy number by real-time PCR

Cells were harvested and genomic DNA was extracted by standard methods using SDS and proteinase K. Real-time PCR was performed on a StepOnePlus^™^ system (Applied Biosystems) with Thunderbird^™^ qPCR Mix (Toyobo) and gene-specific primers (19 to 24 nt) for human or mouse genomic *GAPDH* gene, SRα promoter that drives *BSR* in the plasmid, G5 sequence, and λ-phage (S1 Table). The amount (ng) of each sequence in test DNA was obtained from the standard curve that was obtained for each primer set and standard DNA. For the standard DNA, serially diluted PCR product was used. The copy numbers of SRa promoter, G5, and λ-phage per cell were obtained by dividing the amount of each sequence by that of *GAPDH*.

## Results

### Co-transfection with a core IR-repeat dramatically increased the transformed colony number in both COLO 320DM and CHO DG44 cells

The G5 core IR (971 bp), G5 linked to the AR1 MAR (G5AR1; 2,039 bp in total), or a part of λ-phage (971 bp) was PCR amplified. The product was digested by type I or type II restriction enzymes at sites within the primer sequence, and was ligated to generate either direct or inverted repeats, respectively (Fig 1E and 1F). Self-ligation would normally result in a circular structure at the end of the ligation reaction, although some linear DNA might remain depending on the efficiency of restriction enzyme digestion. Electrophoretic analysis revealed that the most abundant molecules were 2–6 mers (Fig 1G to 1I). Essentially the same results were obtained from more than four independent experiments.

**Fig 2:**
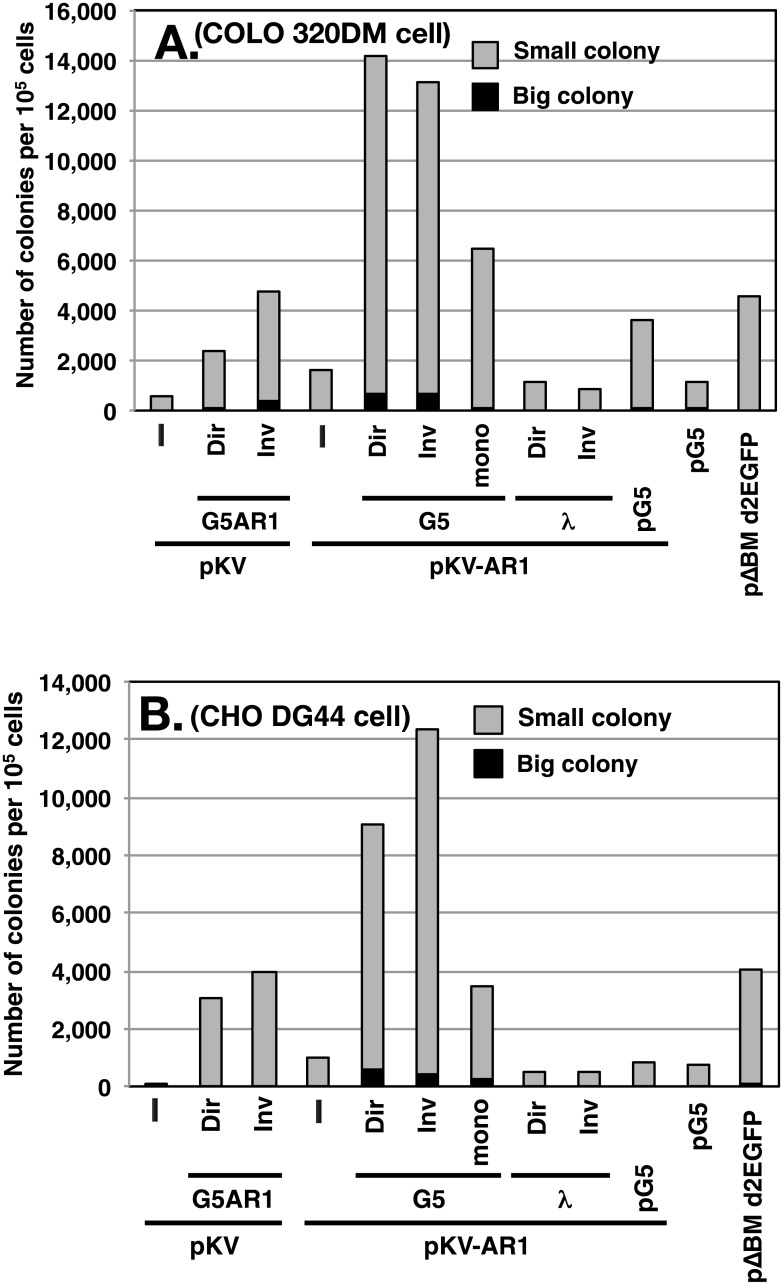
Effect of co-transfected repeat DNA on the transformed colony number. COLO 320DM cells (A) or CHO DG44 cells (B) were transfected with the plasmid pKV or pKV-AR1 or a mixture of the plasmid and the direct (Dir) or the inverted (Inv) repeats of G5AR1, G5, or a part of λ-phage. As a control, pG5 or pΔBM d2EGFP plasmid was transfected. Two days after the transfection, 5 μg/ml blasticidin was added, and the cells were cultured for a further week. The number of large (more than ca. 150 cells) or small (ca. 50 to 150 cells) colonies per 10^5^ transfected cells was counted and plotted. A representative result of three independent experiments that gave similar results is shown.

The direct or inverted repeat DNA was mixed with pKV DNA (Fig 1A) or pKV-AR1 DNA (Fig 1B), and the mixture was transfected into human COLO 320DM or hamster CHO DG44 cells. pKV or pKV-AR1 had *d2EGFP* and *BSR* without or with AR1 MAR, respectively. After selection of the transformants by blasticidin for 1 week, larger numbers of colonies appeared (Fig 2) compared with those from pKV and pKV-AR1 single transfections or the IR/MAR plasmid (pG5 or pΔBM d2EGFP) transfections. The large colony number might reflect the extrachromosomal autonomous replication of the transfected molecule. The effect was reproducibly observed in three independent experiments. The unligated G5 monomer slightly increased the colony number, suggesting that the effect was ligation-dependent. By contrast, the direct or inverted repeats of λ-phage DNA did not affect the colony number (Fig 2), suggesting that the effect was sequence-dependent.

### Co-transfection with a core IR-repeat generated various cytogenetically detectable structures in COLO 320DM cells

**Fig 3:**
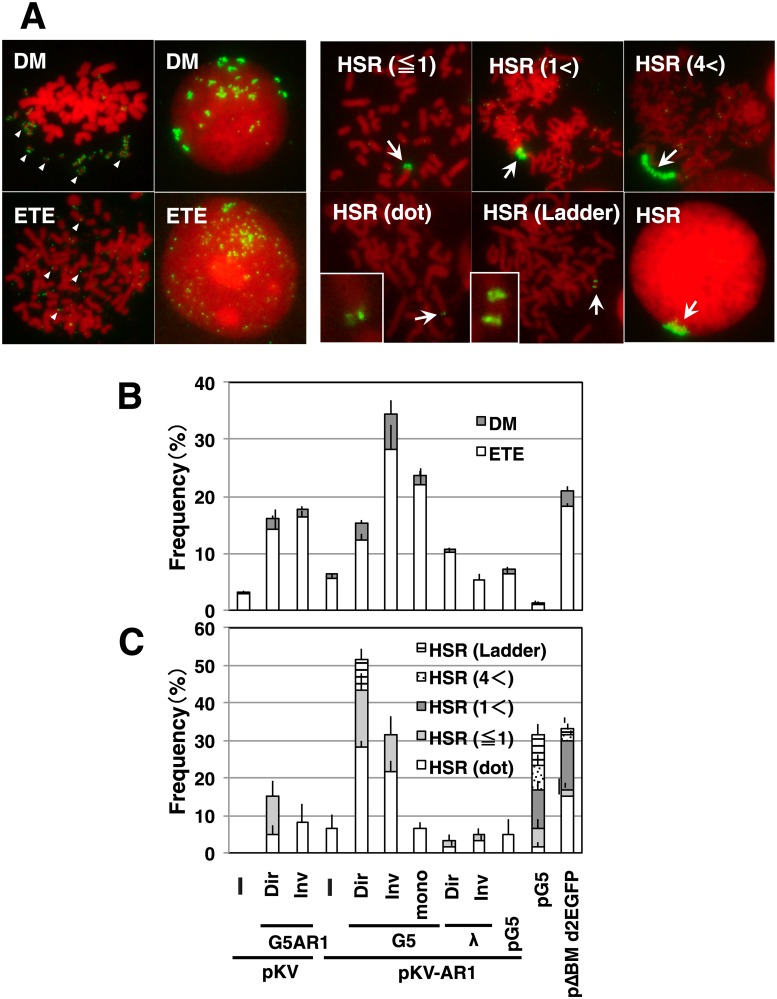
Effect of co-transfected repeat DNA on gene amplification in COLO 320DM cells. After single transfection or co-transfection of the indicated DNA into COLO 320DM cells, the stable transformants were selected by blasticidin for 1 month. The metaphase chromosome spreads from the transformants were analyzed by FISH using a DIG-probe prepared from pG5 plasmid DNA. The hybridized probes were detected by green (FITC) fluorescence, and DNA was counterstained red with propidium iodide. Representative images for each type of amplification are shown in A. The frequency of cells having each type of extrachromosomal (B) or chromosomal (C) amplification was scored by examination of more than 30 metaphase cells in triplicate, and mean +/- standard deviations are plotted.

We prepared metaphase chromosomal spreads from the stable transformants of COLO 320DM cells, and analyzed them by fluorescence in situ hybridization (FISH). The transfected sequence was detected as a bright signal at extrachromosomal DMs, the extrachromosomal tiny elements (ETEs) of smaller signals [20], or chromosomal HSRs of varying length (Fig 3A). The frequency of these signals is shown in Fig 3B and 3C. The plasmids with IR/MAR (pΔBM d2EGFP or pG5) generated both DMs/ETEs and HSRs at a frequency comparable to that in our previous studies [5, 8]. The plasmid without an IR/MAR (pKV) was not amplified, whereas the plasmid with only a MAR (pKV-AR1) generated very short HSRs at a low frequency. On the other hand, co-transfection with pKV/G5AR1 or pKV-AR1/G5 repeats resulted in the appearance of both DMs/ETEs and HSRs at a high frequency. The appearance of HSRs was both ligation- and sequence-dependent, because only a few HSRs appeared in the cells transfected with G5 monomer or λ-phage repeat. Interestingly, longer HSRs occurred more frequently in the cells transfected with G5 direct repeats than in those transfected with inverted repeats, whereas DMs/ETEs were more frequent in cells transfected with inverted repeats than in those transfected with direct repeats. This was evident from the analysis of both metaphase (Fig 3) and interphase cells (S1 Fig). Essentially the same result was obtained from two independent transfections.

### The expression plasmid was embedded in a long array of core IRs in COLO 320DM cells

**Fig 4:**
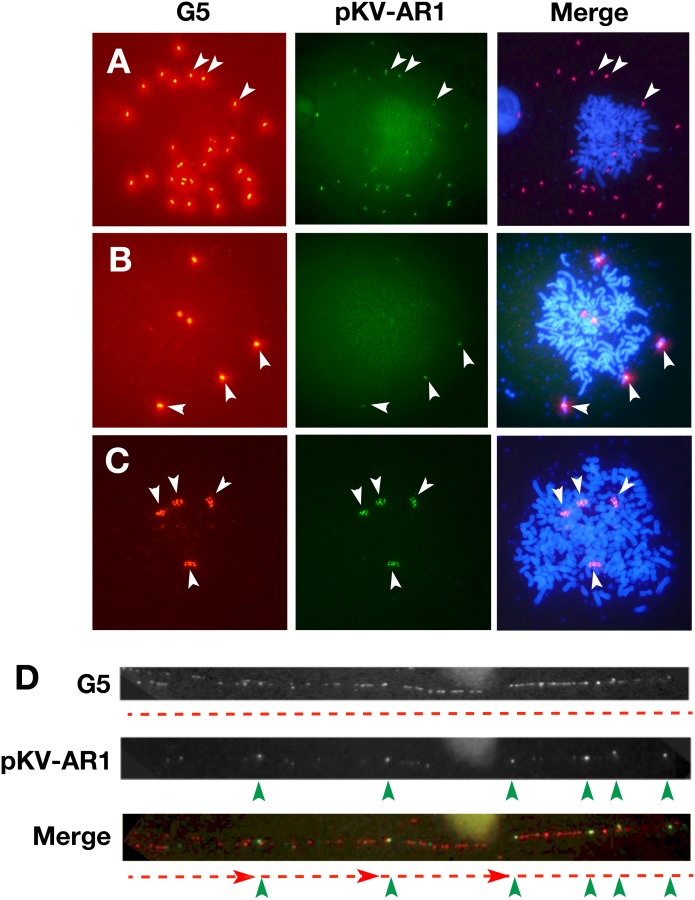
Simultaneous detection of co-transfected sequences in COLO 320DM cells. A mixture of pKV-AR1 DNA and G5 inverted (A and B) or direct (C and D) repeat DNA was co-transfected into COLO 320DM cells, and the stable transformants were selected by blasticidin for a month. Metaphase chromosome spreads (A to C) or chromatin fibers (D) were prepared from these cells. The slides were simultaneously hybridized with biotin-labeled G5 probe and DIG-labeled pKV-AR1 plasmid probe, and the hybridized probes were detected by red (Alexa594) or green (FITC) fluorescence, respectively. DNA was counterstained blue with DAPI in panels A to C. Among metaphase chromosomes, strong G5 signals and weak pKV-AR1 signals were co-localized at multiple DMs (A) or HSRs (B and C). In panel D, small pKV-AR1 signals (green arrowheads) were periodically embedded in the long stretch of G5 signals (red broken lines).

We next addressed the relationship between co-transfected sequences, i.e., G5 and pKV-AR1, by applying two-color FISH to the chromosome spread from COLO 320DM transformants. The result suggested that both sequences were always co-localized at either multiple DMs or HSRs in the cells transfected with G5 direct or inverted repeats (Fig 4A to 4C). The signal of G5 was much stronger than that of pKV-AR1, suggesting that the former sequence was more highly amplified at these structures than the latter sequence.

To increase the resolution, we next applied the two-color FISH to a chromatin fiber that was stretched from the nuclei of these cells. A representative image (Fig 4D) shows that the signal for pKV-AR1 appeared periodically within a long stretch of G5 signal. This strongly suggests that pKV-AR1 was embedded in a long array of G5 repeats.

### Amplified plasmid sequences were detected along with a large number of core IR sequences in co-transfected cells

**Fig 5:**
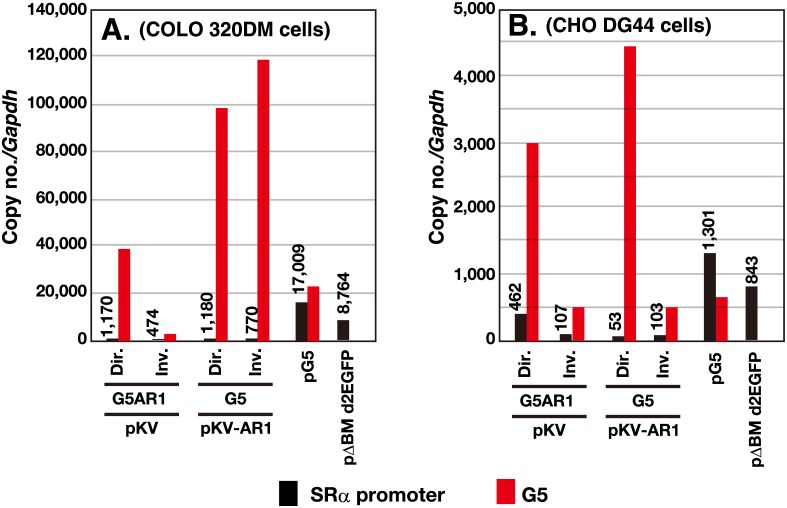
Copy number of the transfected sequence in the stable transformants. After transfection of the indicated DNA into COLO 320DM or CHO DG44 cells, the stable transformants were selected by blasticidin for 1 month. The genomic DNA was isolated and subjected to real-time PCR to determine the amount of SRα promoter, G5, λ-phage, and *Gapdh* sequence. The PCR reaction was done in triplicate, and the mean value was used to calculate the copy number of each sequence relative to *Gapdh* (see graph). The deviation between triplicate reactions was too small to be represented with error bars.

We isolated total DNA from the stable transformants, and estimated the copy number of G5 and plasmid (SRα promoter) relative to genomic *Gapdh* by real-time PCR (Fig 5). The IR/MAR-bearing pΔBM d2EGFP or pG5 was amplified to 8,800 or 17,000 copies, respectively, in COLO 320DM cells, and 843 or 1,300 copies, respectively, in CHO DG44 cells. These numbers might be a few-fold higher than those in previous similar experiments [5, 9]. In general, determination of the absolute copy number by real-time PCR is not accurate. However, in this study, the cells from the pG5 transfection had a similar number of G5 and SRα copies, whereas the cells from pΔBM d2EGFP had only SRα, which was consistent with the structure of the transfected plasmids.

In the cells co-transfected with G5 or G5AR1 direct repeats, a surprisingly large number of G5, i.e., up to 100,000 copies in COLO 320DM cells or around 4,000 copies in CHO DG44 cells, was detected, whereas a much lower copy number of the plasmid was detected. The results of three independent experiments reproducibly revealed a huge excess of the G5 sequence over that in the plasmid. Co-transfection of pKV-AR1 and G5 inverted repeats also resulted in huge amplification of G5 in COLO 320DM. However, the other combinations involving three cases using the inverted repeats resulted in only low levels of G5 amplification. This might reflect the structural instability inherent to the inverted repeats.

### Co-transfected plasmid and core IR-repeat was co-amplified and generated various cytogenetically detectable structures in CHO DG44 cells

**Fig 6:**
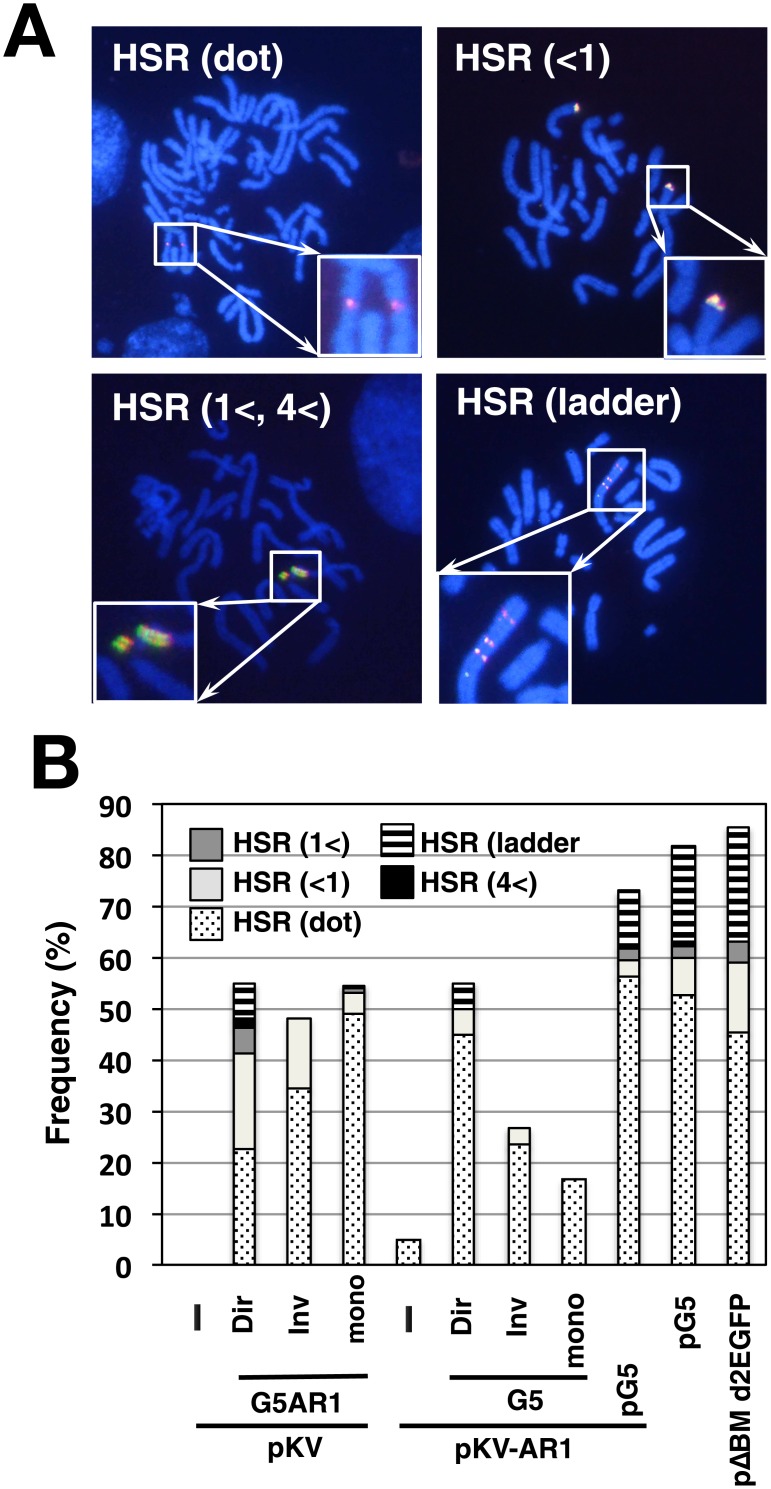
Simultaneous detection of co-transfected sequences in CHO DG44 cells. After transfection of the indicated DNA into CHO DG44 cells, the stable transformants were selected by blasticidin for 1 month, and metaphase chromosome spreads were prepared. The slides were simultaneously hybridized with biotin-labeled G5 probe and DIG-labeled pKV-AR1 plasmid probe, and the hybridized probes were detected by red (Alexa594) and green (FITC) fluorescence, respectively. DNA was counterstained blue using DAPI. Representative images showing various HSRs are shown in A. The frequency of cells having each type of amplification was determined by examining more than 30 metaphase cells with each amplification and is plotted in B.

Our previous study showed that the IR/MAR plasmid generated only a short or a long ladder type HSR in CHO DG44 cells, and it rarely generated extrachromosomal episomes/DMs. Consistently, pG5 or pΔBM d2EGFP generated the former structures at an expected frequency (Fig 6). Furthermore, as expected, pKV (no IR/MAR) or pKV-AR1 (only a MAR) generated no HSRs or the shortest HSRs at a low frequency, respectively. By contrast, we found that the co-transfection of such a plasmid with the G5-AR1 or AR1 repeats resulted in the appearance of FISH signal at large or ladder HSRs. This suggested that such repeats promoted gene amplification also in CHO DG44 cells. Furthermore, simultaneous detection of G5 and plasmid clearly showed that the two sequences were co-amplified in the HSRs (Fig 6A). As in the case of COLO 320DM cells, the signal for G5 was much stronger than the signal for the plasmid, suggesting the higher copy number of the former sequence. This was consistent with the real-time PCR data (Fig 5).

### Co-transfection with core IR-repeats elevated the gene expression from the plasmid, which was sustained for a longer culture

**Fig 7:**
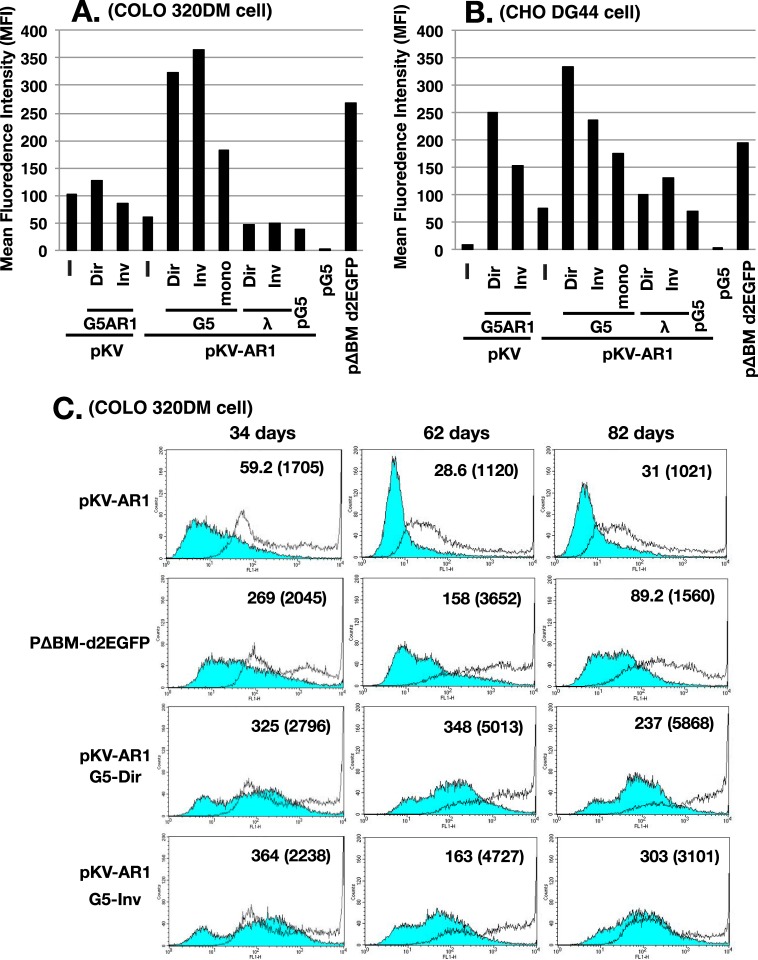
Plasmid-encoded *d2EGFP* expression in the stable transformants. After transfection of the indicated DNA into COLO 320DM (A) or CHO DG44 (B) cells, the stable transformants were selected by blasticidin for 34 or 29 days, respectively. The *d2EGFP* expression was measured using flow cytometry, and the mean fluorescence intensity in arbitrary units was plotted. (C) The indicated COLO 320DM transformants were cultured for 34, 62, and 82 days after transfection, and analyzed by flow cytometry in the absence (blue filled line) or presence (unfilled line) of 2 mM sodium butyrate during the last 3 days. The mean fluorescence intensity for butyrate (-) and (+; parenthesized) culture was noted in each chart.

We next analyzed the *d2EGFP* expression from the plasmid by flow cytometry. Fig 7A and 7B shows the summary of mean fluorescence intensity at about a month after transfection. pG5 did not have *d2EGFP* and served as a negative control. pKV or pKV-AR1 had *d2EGFP*, without or with a MAR, respectively. Compared with these, the IR/MAR-bearing pΔBM d2EGFP showed much higher *d2EGFP* expression in both COLO 320DM and CHO DG44 cells, which reflected the amplification of the plasmid (Figs 3 and 4). Importantly, co-transfection of pKV-AR1 with the G5 direct or inverted repeats greatly increased the *d2EGFP* expression compared with the plasmid alone, in both cell lines (Fig 7A and 7B). The expression level was even slightly higher than that from the standard IR/MAR plasmid, pΔBM d2EGFP. It is important to note that the expression per gene copy was far higher in the former repeat co-transfection method than the latter IR/MAR plasmid method, because the copy number of the plasmid per cell was 8- to 16-fold lower in the former case (Fig 5).

Such high-level expression was also seen in a co-transfection of pKV with the G5AR1 repeats in CHO DG44 cells, but the level was low in COLO 320DM cells (Fig 7). The G5 monomer showed a considerable but lower-level enhancement. This suggests ligation of monomer DNA to multimer in the cell. The λ-phage repeat had hardly any effect, suggesting that the effect was sequence-dependent.

The flow cytometric charts at 34, 62, and 82 days after transfection are shown in Fig 7C. During this period, the average *d2EGFP* expression decreased in cells transfected with pKV-AR1 and pΔBM d2EGFP, which most likely reflected transgene silencing. Sodium butyrate, which inhibits histone deacetylase complexes and partially alleviates the epigenetic silencing, increased the *d2EGFP* expression. Importantly, our data suggested that the culture from the co-transfection of pKV-AR1 and G5 direct or inverted repeats did not show a considerable decrease in the expression. The butyrate also increased the expression in these cultures, suggesting that the amplified transgenes were still silenced in these cells.

## Discussion

**Fig 8:**
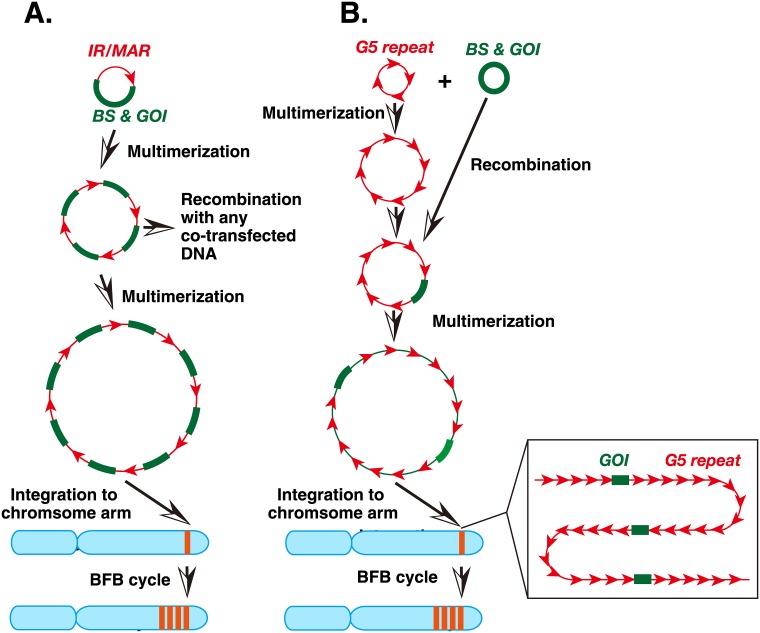
Models that explain amplification of the IR/MAR plasmid (A) or the co-transfected plasmid and G5 repeats (B). (A) The IR/MAR plasmid is maintained and multimerized at an extrachromosomal site. It frequently recombines with co-transfected DNA. After integration of the large plasmid multimer into a chromosome arm, it initiates the BFB cycle, which further amplifies the plasmid sequence and generates a large HSR. (B) G5 is a core IR, and the circular G5 repeat is efficiently multimerized at an extrachromosomal site. It may frequently recombine with the co-transfected plasmid DNA that has the blasticidin-resistance gene (BS) and the gene of interest (GOI). The multimerized large circle may integrate into a chromosome arm, and generate a HSR after the BFB cycle.

Here, we show that co-transfection of core IR (G5)-repeats and an expression plasmid results in embedding of the plasmid sequence within a long array of G5 sequences. The findings in this study in combination with our previous results suggest the mechanism depicted in Fig 8. The first part of the figure is based on our previous studies on IR/MAR plasmid amplification (Fig 8A and reviewed in [23]). Specifically, the IR/MAR plasmid is maintained at an extrachromosomal site after transfection, and it multimerizes to a circular molecule with direct plasmid repeats. Any co-transfected molecule frequently recombines with the IR/MAR plasmid, suggesting that recombination between the extrachromosomal DNA is frequent. If the plasmid multimer integrates into the chromosome arm, it efficiently initiates the BFB cycle, and generates the HSR.

On the other hand, in this study, we transfected G5-repeat DNA into the cells (Fig 8B). Because G5 is a core IR that efficiently supports gene amplification [11], it will efficiently initiate replication. Indeed, G5 has several elements required for replication initiation. According to our model, the G5 repeat is maintained extrachromosomally and multimerizes as efficiently as the IR/MAR plasmid. It then recombines with the co-transfected plasmid, as in the case of the IR/MAR plasmid. Thus, the extrachromosomal molecule with G5 repeats can acquire the drug-resistance gene as well as the gene of interest. The extrachromosomal maintenance of the drug-resistance gene is consistent with the larger number of transformed colonies observed when we used the G5-repeat DNA (Fig 2). Higher transformation efficiency is associated with autonomous extrachromosomal replication; therefore, the autonomous replication sequence was isolated based on the transformation efficiency in yeast cells [24]. Larger colony numbers from the G5 repeat transfection than the IR/MAR plasmid transfection (Fig 2) suggested that the replication initiation was more efficient in the former case. The extrachromosomal maintenance of the transfected sequence was also evidenced by the appearance of DMs/ETEs (Fig 3), as well as the integration of the transfected sequence into many chromosomal sites (Fig 4). Consequently, a large extrachromosomal molecule, with the plasmid embedded in a long array of G5 repeats, is generated. This can integrate into the chromosome arm, where it is elongated to a longer HSR by the BFB cycle.

We examined both direct and inverted repeats of G5. The direct repeats showed stable amplification, and reproducibly generated long HSRs. By contrast, the inverted repeats generated only short HSRs, and generated extrachromosomal DMs or tiny ETEs more frequently than the direct repeats (Fig 3). Furthermore, the copy number of G5 did not show a substantial increase in many cases (Fig 5). This is reasonably explained by the assumption that the inverted repeats are structurally unstable, because they generate a cruciform structure that might be broken by the Holliday junction resolvase [25].

Within the long array of G5 repeats, the expression from the gene of interest was greatly increased. This was especially evident in that the expression per gene copy increased greatly (Fig 7). Furthermore, the high expression level was sustained during long cultivation. We propose that G5 (or G5AR1) favors gene expression and avoids generation of silent chromatin. The CG content of G5 (972 bp) was 0.72%, and that of AR1 MAR (377 bp) was only 0.27%, suggesting that the sequences might have low DNA methylation. DNA methylation is tightly linked to silent chromatin. We recently isolated a human genomic sequence, B-3-31, that prevents silencing of genes amplified by the IR/MAR method [26]. Because there was no core sequence within the 3,271 nt B-3-31, we suggested that the low CG content (0.43%) might prevent gene silencing when it was placed upstream of the promoter. Furthermore, we suggested a model in which the DNA methylation of a few copies of the repeated transgene might initiate silencing, and the silent chromatin generated at this region might spread along the chromatin. Importantly, the long array of G5 repeats is a long stretch of CG-poor sequence that avoids DNA methylation. Furthermore, the IR, in other words the “replicator” of DNA replication or the replication origin, was reported to enhance gene expression [27]. Many reports show that the MAR enhances transgene expression [28–30]. Therefore, there is ample evidence to support the idea that the long stretch of IR (or IR/MAR) provides a superior environment for gene expression.

Gene expression from a repeat sequence, such as transgene repeat, is generally suppressed by a mechanism called repeat-induced gene silencing (RIGS). However, our study showed that the G5 repeat provided an environment that supported gene expression (Fig 7). On the other hand, the λ-phage repeat did not provide such an environment, as evidenced by the low level of gene expression. A previous report showed that the artificial repeat of centromeric alphoid-sequence generates CENP-A chromatin that supports kinetochore formation in vivo [31]. Taken together, these results support the idea that the repeat sequence forms either the chromatin with high gene expression, heterochromatin or CENP-A chromatin, depending on the sequence involved. Uncovering the characteristics of its nucleotide sequence will be an important task for future research.

To increase transgene expression, an elegant approach took advantage of the targeted integration of a transgene to a chromosomal site where gene expression is favored [32, 33]. Here, we showed a novel approach, i.e., artificial generation of a chromosomal environment that favors gene expression.

## Supporting information

S1 Fig Effect of co-transfected repeat DNA on gene amplification in COLO 320DM cells (examinations on interphase nuclei). After single transfection or co-transfection of the indicated DNA into COLO 320DM cells, the stable transformants were selected by blasticidin for 1 month. The metaphase chromosome spreads from the transformants were analyzed by FISH using a probe prepared from pG5 DNA. The frequency of cells having each type of extrachromosomal (A) or chromosomal (B) amplification was scored by examination of more than 300 interphase nuclei in triplicate, and mean +/- standard deviations are plotted (B), or by examination of more than 600 interphase nuclei (A).
(TIFF)

S1 Table Primers used in this study were summarized and shown in this table. (DOC)
