# Supplementary figures and images for: Amplification of a transgene within a long array of replication origins favors higher gene expression in animal cells
Source: PLoS One. 2017 Apr 12;12(4):e0175585. doi: 10.1371/journal.pone.0175585 (PMC5389822; doi:10.1371/journal.pone.0175585)

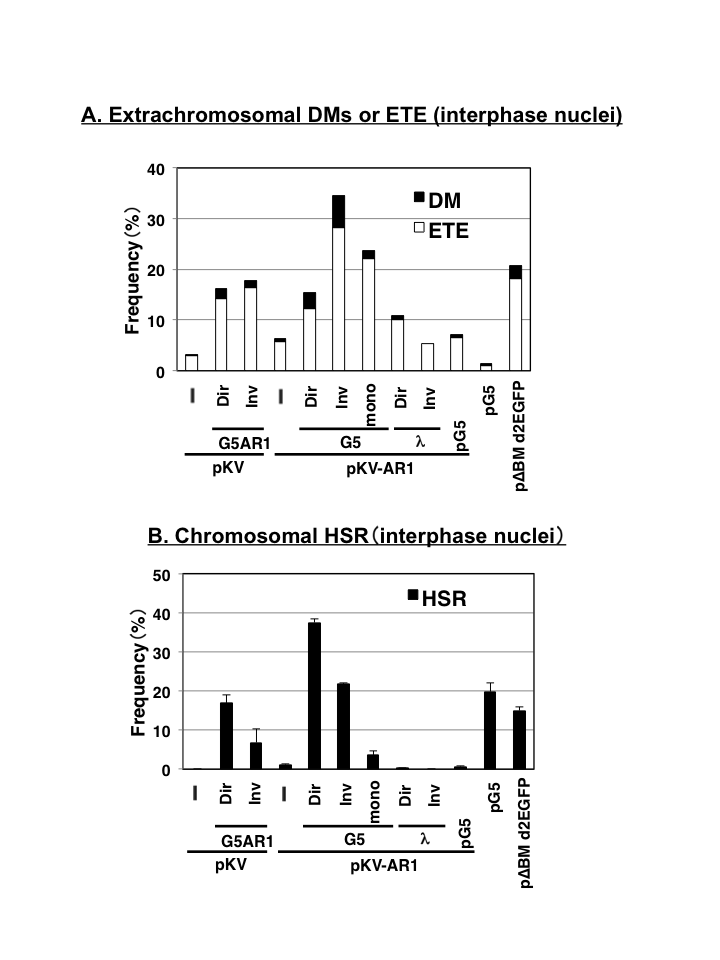

Supplement: S1 Fig — After single transfection or co-transfection of the indicated DNA into COLO 320DM cells, the stable transformants were selected by blasticidin for 1 month. The metaphase chromosome spreads from the transformants were analyzed by FISH using a probe prepared from pG5 DNA. The frequency of cells having each type of extrachromosomal (A) or chromosomal (B) amplification was scored by examination of more than 300 interphase nuclei in triplicate, and mean +/- standard deviations are plotted (B), or by examination of more than 600 interphase nuclei (A). (TIFF) [file pone.0175585.s001.tiff]
