# Supplementary material for: Amplification of a transgene within a long array of replication origins favors higher gene expression in animal cells
Source: PLoS One. 2017 Apr 12;12(4):e0175585. doi: 10.1371/journal.pone.0175585 (PMC5389822; doi:10.1371/journal.pone.0175585)
Supplement: S1 Table — (DOC) [file pone.0175585.s002.doc]

**A. Primers used for the repeat DNA preparation**

G5 Direct repeat

#27 Forward primer; 5’−CATGCTTCGGACCGgcattttctttgacccagga−3’（34 mer）

#28 Reverse primer; 5’−ATGCCAACGGTCCGcccatccccctgtacttttt−3’（34 mer）

G5 Inverted repeat

#29 Forward primer; 5’−CATGCTTACGCGTgcattttctttgacccagga−3’（33 mer）

#30 Reverse primer; 5’−ATGCCAAGTCGACcccatccccctgtacttttt−3’（34 mer）

λ Direct repeat

λ Dir repeat Forward primer;
5’−CATGCTTCGGACCGgcagcgcaacacccttatct−3’（34 mer）

λ Dir repeat Reverse primer;
5’−AGCTGTCCGGTCCGcagctttcctcacccggccc−3’（34 mer）

λ Inverted repeat

λ Inv repeat Forward primer;
5’−CATGCTTACGCGTgcagcgcaacacccttatct−3’（33 mer）

λ Inv repeat Reverse primer;

5’−AGCTGTCGTCGACcagctttcctcacccggccc−3’（34 mer）

G5AR1 Direct repeat

#31 Forward primer; 5’−CATGCTTCGGACCGttggttatgccggtactgcc−3’（34 mer）

#28 Reverse primer; 5’−ATGCCAACGGTCCGcccatccccctgtacttttt−3’（34 mer）

G5AR1 Inverted repeat

#32 Forward primer; 5’−CATGCTTACGCGTttggttatgccggtactgcc−3’（33 mer）

#30 Reverse primer; 5’−ATGCCAAGTCGACcccatccccctgtacttttt−3’（34 mer）、

**B. Primers used in the real-time PCR for gene quantification**

**Human GAPDH**

**Forward primer;** TACTAGCGGTTTTACGGGCG

**Reverse primer;** TCGAACAGGAGGAGCAGAGAGCGA

**Hamster GAPDH**

**Forward primer** ; ACCCAGAAGACTGTGGATGG

**Reverse primer** ; GGATGCAGGGATGATGTTCT

**SRα promoter**

**Forward primer;** CTCGCATCTCTCCTTCACG

**Reverse primer;** CGGTCTCGACCTGAGCTTTA

**G5**

**Forward primer;** GAGCTGAAAGGAAGAAGTAGGAG

**Reverse primer;** AGCGTAGGGTCTCAGTGTTC

**Lambda phage**

**Forward primer;** AGCAGGAGCTGGACTTTACTGATG

**Reverse primer;** GTACGCTGTCCAGATGTGAAAGGT
